# Obstetric Brachial Plexus Palsy and Functional Implications: Which Joint in the Upper Extremity Is More Closely Associated?

**DOI:** 10.3390/medicina60111850

**Published:** 2024-11-10

**Authors:** Gülsena Utku Umut, Zeynep Hoşbay, Müberra Tanrıverdi, Güleser Güney Yılmaz, Okyar Altaş, Alperen Korucu, Atakan Aydın

**Affiliations:** 1 Department of Physiotherapy and Rehabilitation, Faculty of Health Sciences, Biruni University, 34015 Istanbul, Turkey; gulsenautkuumut@gmail.com; 2 Department of Physiotherapy and Rehabilitation, Faculty of Health Sciences, Bezmialem Vakıf University, 34093 Istanbul, Turkey; 3 Department of Occupational Therapy, Faculty of Health Sciences, Hacettepe University, 06100 Ankara, Turkey; 4 Department of Orthopaedics and Traumatology, Hand Surgeon, Başakşehir Çam and Sakura City Hospital, 34480 Istanbul, Turkey; 5 Department of Orthopaedics and Traumatology, Orthopedic Surgeon, Silivri State Hospital, 34570 Istanbul, Turkey; 6 Department of Plastic and Reconstructive Surgery, Faculty of Medicine, İstanbul University, 34093 Istanbul, Turkey

**Keywords:** obstetric brachial plexus palsy, tendon transfer, functionality, body perception

## Abstract

*Background and Objectives:* The objective of this study is to examine the correlation between the active range of motion (ROM) of the affected upper extremity and functional capacity in children with Obstetric Brachial Plexus Palsy (OBPP) who have undergone the modified Hoffer tendon transfer technique. *Materials and Methods:* The study cohort comprised 52 children with OBPP, aged 4–14 years, who had undergone a shoulder tendon transfer. The ROM was quantified using a goniometer, while functionality was evaluated through the administration of the Brachial Plexus Outcome Measure (BPOM). *Results:* The study identified significant correlations between the shoulder ROM and the Brachial Plexus Outcome Measure (BPOM). Specifically, shoulder flexion (*p* = 0.017; r = 0.351) was positively associated with shoulder functionality, while shoulder internal rotation (*p* = 0.001; r = 0.481) was linked to appearance scores. A significant negative relationship was observed between elbow extension (*p* < 0.001; r = −0.512) and elbow and forearm activities. *Conclusions:* The study highlights the necessity of assessing both joint range of motion and body perception for effective treatment and follow-up, to improve the functionality and quality of life for children with OBPP.

## 1. Introduction

Obstetric Brachial Plexus Palsy (OBPP) is defined as varying degrees of paralysis of the C4-T2 radix, trunk, and brachial plexus segments. It is the result of a closed nerve stretch injury involving maternal, obstetric, and fetal factors that exert traction on the anatomically vulnerable plexus during delivery [1,2,3,4]. OBPP presents clinically with a range of symptoms, including shoulder muscle weakness, soft tissue contractions, progressive glenohumeral joint deformities and/or instability, and scapular dyskinesis. The specific manifestations and severity of these symptoms depend on the type and extent of the underlying involvement [5,6]. Since the injury is also a peripheral nerve lesion, sensory and autonomic disorders are also seen in addition to motor problems [7]. Children may also experience motor control disorders [8], sensory losses [9], and developmental delays [10,11]. As a result of the impairment to the functionality of the upper extremity, children experience difficulties in engaging in activities that require fine motor skills, such as personal care (dressing, grooming, etc.), sports, writing, education, and social interactions [12,13]. It is essential to conduct objective and integrated assessments at appropriate time intervals to facilitate the formulation of meaningful clinical decisions and the development of an effective treatment plan for the follow-up of OBPP. The study resulted in an international consensus on assessment methods, and our opinions were expressed. The most commonly used and accepted valid tools are a range of motion measurements, muscle strength measurements, the modified Mallet Scale, and the Brachial Plexus Outcome Measurement [14].

One of the primary objectives of treatment in OBPP is to enhance the child’s functionality [15]. The initial treatment option is conservative treatment. In instances where functional impairment is observed during conservative treatment, early surgical interventions are employed, including nerve transfers, nerve grafts, neurolysis, and analogous techniques [16,17]. Secondary surgical procedures, such as tendon transfers and osteotomies, are also utilized [18]. While there is no definitive algorithm for surgical treatments, clinical developments have demonstrated the safety of these interventions [19].

The most common secondary surgical treatment for OBPP is the modified Hoffer tendon transfer technique, which is used to increase shoulder abduction and external rotation. In this technique, the Latissimus Dorsi and Teres Major tendons are transferred to the greater tubercle of the humerus. The efficacy of this technique in enhancing upper extremity functionality has been substantiated by evidence indicating an increase in the range of motion of the shoulder joint [20,21]. However, following the surgical procedure, it was observed that the patients did not demonstrate a preference for using their extremities, despite the functional recovery of their upper extremities [22]. Also, in our last study, it was seen that although the muscle strength of the affected upper extremity was greater than the unaffected extremity, the children did not prefer to use their affected arm, and the greater muscle strength was not reflected in the function [23]. For this reason, we aimed to investigate the relationship and determinants between the affected upper extremity’s active range of motion degrees and upper extremity functions in children with OBPP treated with the modified Hoffer tendon transfer technique in this study. We hypothesized that the range of motion of children’s postoperative shoulder, elbow, and wrist joints affects the performance of functional movements associated with these joints to varying degrees.

## 2. Materials and Methods

This observational study was conducted between 10 July and 16 August 2024 at Biruni University Physiotherapy and Rehabilitation Department following the Strengthening the Reporting of Observational Studies in Epidemiology (STROBE) checklist [24].

### 2.1. Participants

A total of 52 children with OBPP, aged 4–14 years, who had undergone a shoulder tendon transfer at least six months prior and who had not undergone any elbow surgery, were included in the study. Demographic data were obtained from parents via verbal interview, while clinical data were extracted from patient records, and assessments were conducted by researchers. All evaluations of the children were made at an average of 17.2 ± 0.41 months after surgery.

### 2.2. Outcome Measures

#### 2.2.1. Range of Motion (ROM) Measurements

Range of motion measurements were completed using a universal goniometer by the standards specified by the American Association of Orthopaedic Surgeons (AAOS) [25]. Shoulder abduction and flexion measurements were performed in the standing position to prevent compensatory movements and to observe the spine. Shoulder external and internal rotation measurements were performed in the prone position to stabilize the scapula. Elbow flexion and extension measurements were performed in the supine position. Forearm pronation and supination, wrist flexion and extension measurements were performed in the sitting position. Before the measurements, the desired movements were demonstrated in the unaffected extremity of the child, and then the child was asked to perform the same movement with the affected extremity. All measurements were repeated three times, and the average value of the three measurements was recorded [26].

#### 2.2.2. Functional Assessment

The Brachial Plexus Outcome Measure (BPOM) was used for the functional assessment. The scale was developed by Ho et al. to evaluate the functionality and participation in daily life of children with OBPP [27]. The scale has two subscales: Activity and Self-Assessment. The BPOM Activity subscale consists of 3 parts: shoulder, elbow and forearm, wrist, finger and thumb, and a total of 11 items. Scoring is performed between one and five. High scores are associated with good functionality. The BPOM Self-Assessment subscale consists of two visual analog scales that evaluate the child’s arm and hand functioning, and one visual analog scale that evaluates the appearance of the child’s arm and hand. The BPOM-TR, which had a reliability of 0.938 and was translated into Turkish by Hoşbay et al. was used in the study [28].

All assessments were made for the affected side of the child.

### 2.3. Statistical Analysis

Statistical analysis was performed using the “SPSS 22.0 for Windows” statistics program. In descriptive statistical analyses, arithmetic mean ± standard deviation (mean ± SD) was used, and categorical variables were shown as percentages (%). “Shapiro–Wilks test” was used to determine whether the distribution of continuous variables was normal or not. Active ROM degrees for upper extremity joints of children with OBPP were given as mean, standard deviation, and minimum–maximum. The relationship between goniometric measurement and BPOM was analyzed using “Pearson Correlation Analysis”. It was interpreted as 0.00–0.19 = very weak; 0.20–0.39 = weak; 0.40–0.59 = moderate; 0.60–0.79 = strong; 0.80–1.00 = very strong correlation. The statistical significance level was accepted as *p* < 0.05.

## 3. Results

Twenty girls (38%) and thirty-two boys (62%) with OBPP, with an average age of 8.66 ± 2.40, participated in the study. Demographic and clinical characteristics of the cases are given in Table 1.

The shoulder joint mean active abduction was 122.71 (±39.82), active flexion was 135.43 (±37.78), active external rotation was 64.72 (±21.11), and active internal rotation was 19.34 (±24.34) degrees. The elbow joint mean active flexion was 122.88 (±21.67) and active extension was −9.33 (±18.63) degrees. Forearm active pronation was 43.06 (±32.37) and supination was 46.63 (±29.85) degrees. The wrist joint mean active flexion was 60.51 (±27.76) and active extension was 43.40 (±19.31) degrees. The upper extremity ROM measurements of the cases are given in Table 2.

The relationship between upper extremity ROM measurements and the BPOM results is given in Table 3.

A significant positive relationship was found between the BPOM-shoulder activity scores and the shoulder active flexion ROM measurement (*p* = 0.017; r = 0.351). Also, a significant relationship was found between the BPOM-shoulder appearance scores and the shoulder active internal rotation ROM measurement (*p* = 0.001; r = 0.481). No significant relationship was found between the BPOM-self-evaluation scores and the shoulder ROM measurements (*p* > 0.05) (Table 3).

A significant negative relationship was detected between the BPOM-elbow and forearm activity scores and the elbow active extension (*p* < 0.001; r = −0.512), the forearm active pronation (*p* = 0.014; r = 0.339), the forearm active supination (*p* = 0.014; r = 0.439) ROM measurements. A significant positive relationship was detected between BPOM-elbow and forearm appearance scores and the forearm active pronation ROM measurement (*p* = 0.001; r = 0.446). Also, a significant positive relationship was detected between the BPOM-self-evaluation scores and the forearm active pronation (*p* = 0.007; r = 0.388), and the forearm active supination (*p* = 0.005; r = 0.403) ROM measurements. No significant relationship was found between the BPOM-appearance scores and BPOM-self-evaluation scores and the elbow ROM measurements (*p* > 0.05).

A significant positive relationship was detected between the BPOM-wrist, finger, and thumb activity scores and the wrist active flexion (*p* = 0.005; r = 0.403), and wrist active extension (*p* = 0.007; r = 0.488) ROM measurements. Also, a significant positive relationship was detected between the BPOM-self-evaluation scores and the wrist active flexion (*p* = 0.014; r = 0.339), and the wrist active extension (*p* < 0.001; r = 0.639) ROM measurements. No significant relationship was found between the BPOM-appearance scores and the wrist ROM measurements (*p* > 0.05).

## 4. Discussion

In this study, we aimed to investigate the relationship and determinants between the upper extremity’s active ROM degrees and upper extremity functions in children with OBPP treated with the modified Hoffer tendon transfer technique. The findings of our study indicate a correlation between upper extremity joint ROM and functional outcomes in children with OBPP who have undergone shoulder tendon surgery.

The structural problems seen in the upper extremity in OBPP limit functionality and participation in daily living activities. Although both conservative and surgical treatments aim to increase the child’s functionality; to the best of our knowledge, there are limited studies examining structural factors affecting functionality in OBPP [29]. The follow-up of children with OBPP is typically conducted with an emphasis on the level of impairment, encompassing assessments of muscle strength, ROM, and sensory evaluations [17,18]. However, these evaluations do not provide a comprehensive assessment of the functional status of the upper extremity, participation in daily life, or the child’s perceptions of the disability. Considering that the effects of OBPP are not limited to body structures but affect various aspects of the individual’s well-being, the use of tools that evaluate the patient from a biopsychosocial perspective becomes important. Brown et al. suggested the use of BPOM to create a global consensus in the evaluation of children with OBPP [30]. The BPOM, an ICF-based assessment tool, with which the Turkish validation study was performed by our researchers, was used in our study. The BPOM is a tool used to evaluate the quality of movement in 5 grades. It may be challenging to differentiate between a score of 3 (describing passive movements) and a score of 4 (describing minimal compensatory movements) during the evaluation process. However, this did not constitute a limitation, given that the evaluations were conducted by researchers with extensive experience in OBPP. It was clinically observed that children with higher functional activity scores had lower self-evaluation scores.

There are many studies in the literature about the results of shoulder tendon transfers using the modified Hoffer technique to increase shoulder abduction and external rotation in children with OBPP. While there is an increase in shoulder abduction and external rotation after the modified Hoffer technique, limitations are seen in internal rotation. Previous studies have highlighted gains in external rotation; however, evidence on the consequences of losses in internal rotation is limited [31,32]. It has been stated that routine evaluation of perceived physical appearance may be useful in identifying people who may benefit from this treatment [30]. Adidharma et al. stated that losses after shoulder tendon transfer were not reflected in BPOM scores [33]. The findings of our study indicate a correlation between active flexion movement and activities, as well as active internal rotation movements and the appearance of the shoulder. As previously stated in the literature [31,32], after the shoulder tendon transfer surgery, shoulder IR degrees were affected in our cases. Although this limitation was not reflected in the activity scores, it was assumed that the children scored the discomfort they felt due to this IR limitation. On the other hand, we think that the facts that shoulder flexion limitation is reflected in the activity score, and IR limitation is reflected in the appearance, and there is no relationship with other limitations, are due to children evaluating their functions depending on the function of the distal joints.

The elbow joint plays an important role in maintaining stability during various daily living activities, including lifting, carrying, pushing, and pulling. A lack of full elbow extension negatively affects the position of the hand and functions. Elbow flexion contractures are common in children with OBPP. In addition to the functional effects of these contractures, they also have psychosocial effects due to their appearance. These effects may be affected by the personal factors of the child and his/her family, as well as the degree of contracture [34,35]. In addition to evaluating children’s body structures and functionality, it is also considered important to evaluate their perceived appearance in terms of surgical and rehabilitative treatment planning [36]. In accordance with the findings of previous research, it was observed that active extension movements of the elbow were related to elbow activities in the study. Approaching the neutral position in extension degrees increased activity scores. Conversely, there was no relationship between elbow movements and appearance or self-evaluation scores. Children with OBPP may perceive elbow flexion contractures as a relatively minor problem compared to other damage to their upper extremities. It has been stated that children with elbow flexion contractures greater than thirty degrees do not report any functional limitations regarding the functionality of their daily activities. It is thought that they may have integrated the elbow posture into their body schema and did not perceive this situation as a limitation [37]. Given that the mean values of elbow flexion contractures observed in the children in our study were below 30 degrees, it was concluded that this condition was not reflected in the children’s appearance scores. Since children mostly evaluate the distal joints in terms of appearance and self-evaluation, we think that elbow flexion contractures affect activity but are not related to appearance and self-evaluation. When compared according to elbow flexion degrees, a 30-degree flexion contracture at the elbow may be decisive for appearance and self-evaluation, similar to the study by Ho et al. [36].

There is no consensus regarding the optimal functional position of the forearm. It has been stated that the neutral position or slightly pronation position is the most functional position for writing, while a position between pronation and supination will be more functional for a strong grip [38]. Nevertheless, without patient-centered outcome measures, studies to date have not shed light on the optimal forearm position for function [38]. In studies, the child’s ability to perform activities that require supination, neutral, or pronation of the forearm is evaluated by using a computer mouse, playing drums, and holding a plate, respectively [11]. In our study, the flexion and extension ROM degrees of the wrist, and the pronation and supination ROM degrees of the forearm were found to be related to activity. Pronation was found to be related to all activity, appearance, and self-evaluation scores, whereas supination was only related to activity and self-evaluation scores. After surgery to increase supination ROM degrees, improvements in both functionality and appearance are observed [39]. However, in our study, children with OBPP who had undergone forearm surgery were not included, children who had only shoulder surgery were evaluated. It is thought that the degree of supination has a greater impact on activity limitations, and this is reflected in the child’s self-evaluation. It has been reported that the forearm, which is more pronated compared to the beggar’s hand, is perceived to have a closer to normal appearance [39,40,41]. Given that the mean supination degree for the children in our study was 46 and the mean pronation degree was 43, the forearm is thought to be in a position close to neutral, which is accepted as the reason why it is not reflected in the appearance scores.

### 4.1. Clinical Implications

The evaluation of the upper extremity’s range of motion following surgical procedures in children with OBPP may provide insights into the functional capabilities of these children for clinicians. It should be noted that the range of motion is not solely associated with functionality; it may also influence children’s body perception. Furthermore, in addition to objective measurement methods, it would be useful to include self-reported measurements in clinical practice.

### 4.2. Limitations

The inability to group children in terms of age, type of involvement, and degree of contracture represents a limitation in the generalizability of the results. Also, the absence of passive range of motion values represents another limitation. It is important to consider that this study was retrospective when evaluating its results. To confirm and generalize the results, prospective studies with larger sample groups and the same outcome measures are required.

## 5. Conclusions

There is a relationship between the upper extremity joints’ range of motion and functionality in children with OBPP who have undergone shoulder tendon surgery. Children’s shoulder flexion range of motion and their functionality related to these movements, shoulder internal rotation range of motion, and self-perceived appearance are associated with each other. In addition, children’s elbow extension range of motion affects their activities related to the elbow and forearm. We would like to emphasize that evaluating children’s body perception is also important for treatment and follow-up.

## Figures and Tables

**Table 1:** Demographic and clinical data of children.

	Mean ± SD N (%)	min–max
Age (year)	8.66 ± 2.40	4.5–14
Gender		
Girl	20 (38%)	
Boy	32 (62%)	
Birth weight (gram)	4054 ± 86	2840–5350
Affected side		
Right	32 (61.53%)	
Left	20 (38.47%)	
Involvement		
C5–C6	9 (17.3%)	
C5–C7	25 (48%)	
Total	18 (34.7%)	
Surgical treatment		
Primary surgery + tendon transfer	9 (17.4%)	
Tendon transfer	43 (82.6%)	
Accompanying injuries		
Horner	4 (7.7%)	
Torticollis	1 (1.9%)	
None	47 (90.3%)	
Mallet scores	15.44 ± 1.51	12–18

SD: Standard deviation; min: minimum; max: maximum.

**Table 2:** Upper extremity’s range of motion degrees.

ROM (Degrees)		Mean ± SD	min–max
SHOULDER	Abduction	122.71 ± 39.82	20.00–180.00
	Flexion	135 ± 37.78	20.00–180.00
	External Rotation	64.72 ± 21.11	15.00–90.00
	Internal Rotation	19.34 ± 24.34	−20.00–75.00
ELBOW	Flexion	122.88 ± 21.67	50.00–145.00
	Extension	−9.33 ± 18.63	−55.00–60.00
FOREARM	Pronation	43.06 ± 32.37	−70.00–90.00
	Supination	46.63 ± 29.85	−20.00–90.00
WRIST	Flexion	60.51 ± 27.76	−5.00–90.00
	Extension	43.40 ± 19.31	0.00–90.00

SD: Standard deviation; min: minimum; max: maximum.

**Table 3:** The relationship between upper extremity’s range of motion measurements and BPOM results.

**SHOULDER**	Active Flexion		Active Internal Rotation		Active External Rotation		Active Abduction	
	*p*	r	*p*	r	*p*	r	*p*	r
BPOM-Shoulder	0.017 *	0.351	0.683	0.062	0.556	−0.08	0.761	−0.04
BPOM-Appearance	0.887	0.023	0.001 *	0.481	0.774	0.084	0.779	−0.04
BPOM-Self Evaluation	0.435	−0.18	0.202	−0.22	0.787	−0.04	0.80	−0.26
**ELBOW**	Active Flexion				Active Extension			
	*p*		r		*p*		r	
BPOM-Elbow and Forearm	0.317		−0.142		0.000 *		−0.512	
BPOM-Appearance	0.158		−0.199		0.937		−0.011	
BPOM-Self Evaluation	0.409		−0.117		0.347		0.133	
**FOREARM**	Active Pronation				Active Supination			
	*p*		r		*p*		r	
BPOM-Elbow and Forearm	0.014 *		0.339		0.014 *		0.439	
BPOM-Appearance	0.001 *		0.446		0.277		0.153	
BPOM-Self Evaluation	0.007 *		388		0.005 *		0.403	
**WRIST**	Active Flexion				Active Extension			
	*p*		r		*p*		r	
BPOM-Wrist, Finger, Thumb	0.005 *		0.403		0.007 *		0.488	
BPOM-Appearance	0.567		0.086		0.994		−0.001	
BPOM-Self Evaluation	0.014 *		0.339		0.000 *		0.639	

BPOM: Brachial Plexus Outcome Measure; * Pearson Correlation Analysis, *p* < 0.05.

## Data Availability

No clinical trial registration was obtained because our study consists of assessments included in routine checks at the clinic. And there was no intervention within the scope of the study.

## References

[1] Van der Looven R., Le Roy L., Tanghe E., Samijn B., Roets E., Pauwels N., Deschepper E., De Muynck M., Vingerhoets G., Van den Broeck C. (2020). Risk factors for neonatal brachial plexus palsy: A systematic review and meta-analysis. Dev. Med. Child. Neurol..

[2] Chauhan S.P., Blackwell S.B., Ananth C.V. (2014). Neonatal brachial plexus palsy: Incidence, prevalence, and temporal trends. Semin. Perinatol..

[3] Lagerkvist A.L., Johansson U., Johansson A., Bager B., Uvebrant P. (2010). Obstetric brachial plexus palsy: A prospective, population-based study of incidence, recovery, and residual impairment at 18 months of age. Dev. Med. Child Neurol..

[4] Dumontier C., Gilbert A. (1990). Traumatic brachial plexus palsy in children. Ann. Chir. Main. Memb. Super..

[5] Van der Looven R., Hermans L., Coupe A.M., De Muynck M., Vingerhoets G. (2022). Neonatal brachial plexus palsy and hand representation in children and young adults. Dev. Med. Child. Neurol..

[6] Medeiros D.L., Agostinho N.B., Mochizuki L., Oliveira A.S. (2020). Qualıty of Life and Upper Limb Function of Children with Neonatal Brachial Plexus Palsy. Rev. Paul. Pediatr..

[7] Pondaag W., Malessy M.J., van Dijk J.G., Thomeer R.T. (2004). Natural history of obstetric brachial plexus palsy: A systematic review. Dev. Med. Child. Neurol..

[8] Frade F., Gómez-Salgado J., Jacobsohn L., Florindo-Silva F. (2019). Rehabilitation of Neonatal Brachial Plexus Palsy: Integrative Literature Review. J. Clin. Med..

[9] Corkum J.P., Kuta V., Tang D.T., Bezuhly M. (2017). Sensory outcomes following brachial plexus birth palsy: A systematic review. J. Plast. Reconstr. Aesthet. Surg..

[10] Buitenhuis S., van Wijlen-Hempel R.S., Pondaag W., Malessy M.J. (2012). Obstetric brachial plexus lesions and central developmental disability. Early Hum. Dev..

[11] Strömbeck C., Remahl S., Krumlinde-Sundholm L., Sejersen T. (2007). Long-term follow-up of children with obstetric brachial plexus palsy II: Neurophysiological aspects. Dev. Med. Child. Neurol..

[12] Alyanak B., Kılınçaslan A., Kutlu L., Bozkurt H., Aydın A. (2013). Psychological adjustment, maternal distress, and family functioning in children with obstetrical brachial plexus palsy. J. Hand Surg. Am..

[13] Strömbeck C., Fernell E. (2003). Aspects of activities and participation in daily life related to body structure and function in adolescents with obstetrical brachial plexus palsy: A descriptive follow-up study. Acta Paediatr..

[14] Pondaag W., Malessy M.J.A. (2018). Outcome assessment for Brachial Plexus birth injury. Results from the iPluto world-wide consensus survey. J. Orthop. Res. Off. Publ. Orthop. Res. Soc..

[15] Matthews D.J. (2018). Function of the unaffected arms of children with neonatal brachial plexus injuries. Eur. J. Paediatr. Neurol..

[16] Coroneos C.J., Voineskos S.H., Coroneos M.K., Alolabi N., Goekjian S.R., Willoughby L.I., Thoma A., Bain J.R., Brouwers M.C., Canadian OBPI Working Group (2015). Primary Nerve Repair for Obstetrical Brachial Plexus Injury: A Meta-Analysis. Plast. Reconstr. Surg..

[17] Socolovsky M., Lovaglio A., Bonilla G., Masi G.D., Barillaro K., Malessy M. (2023). Brain plasticity and age after restoring elbow flexion with distal nerve transfers in neonatal brachial plexus palsy and non neonatal traumatic brachial plexus injury using the plasticity grading scale. J. Neurosurg..

[18] Buterbaugh K.L., Shah A.S. (2016). The natural history and management of brachial plexus birth palsy. Curr. Rev. Musculoskelet. Med..

[19] Li H., Chen J., Wang J., Zhang T., Chen Z. (2023). Review of rehabilitation protocols for brachial plexus injury. Front. Neurol..

[20] Russo S.A., Nice E.M., Chafetz R.S., Richards J.G., Zlotolow D.A., Kozin S.H. (2024). Impact of tendon transfer on scapulothoracic and glenohumeral motion in children with brachial plexus birth injuries. J. Shoulder Elbow Surg..

[21] Ozkan T., Okumus A., Aydin A., Ozkan S., Tuncer S. (2005). Brachioradialis transposition for elbow extension in obstetrical brachial plexus palsy. Tech. Hand Up. Extrem. Surg..

[22] Bicer A., Aydın A., Özkan T., Özkan S., Hosbay Z., Berköz Ö. (2022). Transfer Of Brachıalıs Muscle To Trıceps For Restoration of Elbow Extension In Patients with Obstetrıc Palsy Sequelae. J. Istanb. Fac. Med..

[23] Hosbay Z., Utku Umut G., Tanrıverdi M., Altas O., Aydin A. (2024). Effect of Muscle Strength on Functionality after Shoulder Tendon Transfer in Brachial Plexus Birth Injury: Is There a Relationship between Them?. Children.

[24] von Elm E., Altman D.G., Egger M., Pococck S.J., Gøtzsche P.C., Vandenbroucke J.P. (2008). The Strengthening the Reporting of Observational Studies in Epidemiology (STROBE) statement: Guidelines for reporting observational studies. J. Clin. Epidemiol..

[25] Hunsaker F.G., Cioffi D.A., Amadio P.C., Wright J.G., Caughlin B. (2002). The American academy of orthopedic surgeons outcomes instruments: Normative values from the general population. J. Bone Joint Surg. Am..

[26] Otman A.S., Köse N. (2019). Tedavi Hareketlerinde Temel Değerlendirme Prensipleri.

[27] Ho E.S., Curtis C.G., Clarke H.M. (2012). The brachial plexus outcome measure: Development, internal consistency, and construct validity. J. Hand Ther..

[28] Hosbay Z., Ozkan S., Tanriverdi M., Aydin A. (2019). Reliability and validity of the Brachial Plexus Outcome Measure in children with obstetric brachial plexus palsy. J. Hand Ther..

[29] de Moraes A.A., Dantas D.S., Chagas A.C.S., de Melo P.H., de Oliveira D.A. (2023). Linking assessment instruments for brachial plexus injury to the international classification of functioning, disability and health. J. Hand Ther..

[30] Brown H., van der Looven R., Ho E.S., Pondaag W. (2024). Patient reported outcomes in brachial plexus birth injury: Results from the iPLUTO world-wide consensus survey. Disabil. Rehabil..

[31] Thamer S., Kijak N., Toraih E., Thabet A.M., Abdelgawad A. (2023). Tendon Transfers to Restore Shoulder Function for Obstetrical Brachial Plexus Palsy: A Systematic Review of the Literature. JBJS Rev..

[32] Safoury Y.A., Eldesoky M.T., Abutaleb E.E., Atteya M.R., Gabr A.M. (2017). Postoperative physical therapy program for latissimus dorsi and teres major tendons transfer to rotator cuff in children with obstetrical brachial plexus injury. Eur. J. Phys. Rehabil. Med..

[33] Adidharma W., Lewis S.P., Liu Y., Osorio M.B., Steinman S.E., Tse R.W. (2020). Shoulder Release and Tendon Transfer following Neonatal Brachial Plexus Palsy: Gains, Losses, and Midline Function. Plast. Reconstr. Surg..

[34] Ho E.S., Parsons J.A., Davidge K., Clarke H.M., Lawson M.L., Wright F.V. (2022). Developing a decision aid for youth with brachial plexus birth injuries facing treatment decisions for an elbow flexion contracture. PM R.

[35] Sheffler L.C., Lattanza L., Hagar Y., Bagley A., James M.A. (2012). The prevalence, rate of progression, and treatment of elbow flexion contracture in children with brachial plexus birth palsy. J. Bone Joint Surg. Am..

[36] Ho E.S., Klar K., Klar E., Davidge K., Hopyan S., Clarke H.M. (2019). Elbow flexion contractures in brachial plexus birth injury: Function and appearance related factors. Disabil. Rehabil..

[37] Dolan P., Kahneman D. (2008). Interpretations of utility and their implications for the valuation of health. Economics.

[38] Rolfe K.W., Green T.A., Lawrence J.F. (2009). Corrective osteotomies and osteosynthesis for supination contracture of the forearm in children. J. Pediatr. Orthop..

[39] Hamdi N.B., Doubi M., Abalkhail T.B., Mortada H. (2022). Clinical and psychosocial outcomes following correction of supination deformity in obstetrical brachial plexus palsy patients: A retrospective study. BMC Musculoskelet. Disord..

[40] Bhardwaj P., Varadharajan V., Salyan S., Venkatramani H., Sabapathy S.R. (2023). Forearm Deformities in Birth Brachial Plexus Palsy—Patient Profile and Management Algorithm. J. Hand Surg. Asian Pac. Vol..

[41] Al-Qattan M.M., Al-Khawashki H. (2002). The, “beggar’s” hand and the “unshakable” hand in children with total obstetric brachial plexus palsy. Plast. Reconstr. Surg..

